# Postpartum Depressive Symptoms and Associated Factors in Married Women: A Cross-sectional Study in Danang City, Vietnam

**DOI:** 10.3389/fpubh.2017.00093

**Published:** 2017-04-27

**Authors:** Thang Van Vo, Thi Kim Duong Hoa, Tuyen Dinh Hoang

**Affiliations:** ^1^Institute for Community Heath Research, Hue University of Medicine and Pharmacy, Hue, Vietnam; ^2^Faculty of Public Heath, Hue University of Medicine and Pharmacy, Hue, Vietnam; ^3^Danang University of Medical Technology and Pharmacy, Danang, Vietnam

**Keywords:** postpartum depression, Edinburgh Postpartum Depression Scale, mental health, pregnancy, gender

## Abstract

**Introduction:**

Postpartum depression (PPD) among women is a common mental health concern. It occurs at a time of major life change, coupled with the increased responsibilities associated with the care of a newborn infant. In Vietnam, the prevalence of depressive symptoms after giving birth has not been fully investigated. Research in the Northern provinces, in Ho Chi Minh City, and in Hue suggests postnatal depressive symptoms among women are common. This research aims to (1) estimate the prevalence of PPD symptoms among married women in one Vietnam city (Danang) and (2) identify the social and personal factors associated with postpartum depressive symptoms.

**Methods:**

This cross-sectional study was conducted from July 2013 to August 2014 in 10 wards of Hai Chau District, Danang. A total of 600 mothers who gave birth 4 weeks to 6 months prior to being interviewed were recruited. Interviews were conducted using structured questionnaires, which included several dimensions: demographics, family living arrangements, expectations of pregnancy, expectations of infant gender, the woman’s relationship with her husband, exercise after birth, infant health, and anxiety about matters other than the birth. The Edinburgh Postpartum Depression Scale (EPDS) was used to examine PPD symptoms, with a cutoff point of 12/13.

**Results:**

EPDS scores indicated the prevalence of PPD symptoms was 19.3% (95% CI: 16.16–22.50). Among women with PPD symptoms, 37.9% had suicidal thoughts in the previous seven days. Multivariate logistic regression indicated that the following key factors were significantly associated with PPD symptoms: Not being able to rely on their husband for help, having a husband who does not spend time to discuss problems, having anxiety about matters other than the birth, not exercising after giving birth, and having an ill baby.

**Conclusion:**

These findings should be interpreted in relation to other recent research in Vietnam. A consistent pattern of prevalence estimates and associated social factors is emerging that has implications for the postpartum care of mothers.

## Introduction

Postpartum depression (PPD) is a common perinatal mental disorder among women, occurring 4 weeks to 1 year after giving birth. Research reveals that PPD is a “silent killer” that contributes to maternal mortality and can have health and developmental consequences for children (1, 2). There is evidence that the prevalence of postpartum mental disorders is around 19.8% in low and lower-middle income countries (3). However, the rates of PPD differ between countries and territories around the world. Klainin and Arthur (4) reported that the rate of PPD ranged from 3.5 to 63% in 17 Asian countries between 1998 and 2008 (4).

An increasing number of epidemiological studies on depression and perinatal mental health have been conducted in Vietnam during the last 10 years. According to mental health surveys conducted in the country, at least 57% of people have never attended a medical appointment where they were screened for common mental disorders (5). Meanwhile, common perinatal mental disorders like PPD receive little attention because they are not screened for, or diagnosed as an illness by many health services. Mothers also rarely participate in mental health screening due to a lack of knowledge about perinatal mental health, limited opportunities for screening, and being afraid of stigmatization if diagnosed (6). A study of common perinatal mental disorders in pregnant and postpartum women in Ha Noi and Ha Nam found the prevalence of PPD was 29.9%, yet no women in the study had ever been diagnosed with a mental disorder, nor received mental health care in the past (7). Similarly, research in Hue in 2013 found that 18.1% of women had symptoms of PPD and very few women had ever been diagnosed with depression (8). Many non-biological risk factors contribute to PPD. Such factors include social and individual factors like poverty, experiencing stressful life events, and the gender dynamic within relationships with intimate partners (9). This research sought to explore social and personal risk factors rather than clinical factors occurring during delivery and the postnatal period. The study was conducted among women in Danang city, as it is one of the most developed cities in Vietnam.

In Vietnam, it is important to develop services to promote the early identification of PPD and to help physicians with identification and treatment of common perinatal mental disorders. Research, such as the study reported here, contributes to the evidence based on common perinatal mental disorders in Vietnam, which can be used to inform services and interventions. For the above reasons, we conducted the study, “*Postpartum depressive symptoms and associated factors in married women: a cross-sectional study in Danang city, Vietnam*” with the following objectives:

1. To estimate the prevalence of postpartum depressive symptoms in married women in one Vietnamese city (Danang).
2. To identify social and personal factors associated with postpartum depressive symptoms.

## Sample and Methods

### Sampling Strategy

This was a cross-sectional prevalence study. Data were collected between July and December of 2013. The research participants were married women who were interviewed 4 weeks to 6 months after giving birth.

The sample size was calculated using the formula “estimating a proportion of the population” (10):

$$n=Z^{2}(1-\alpha /2)\frac{p(1-p)}{d^{2}}=355,$$

in which

– *Z* = value from standard normal distribution corresponding to desired confidence level [*Z*(1 − α/2) = 1.96 for 95% CI].
– *p* was expected true proportion, *p* = 18.1% (0.18), pursuant to the result of PPD rate found by Murray in Thua Thien Hue, Vietnam in 2012 (8).
– *d* = 0.04 (4%) was desired precision.

A two-stage cluster sampling method was applied [probability proportional to size (PPS)]. The sample size was multiplied by the design effect (DEFF). We chose a DEFF = 1.5, which indicated that *n* would be 355 × 1.5 = 532. Consequently, 600 research participants were selected.

The PPS sampling and simple random sampling (SRS) were conducted as follows:

Step 1: All wards in Hai Chau District were numbered. A cumulative frequency table was generated, which produced the total number of married women giving birth from 4 weeks to 6 months in Hai Chau District, identified as *m* (*m* = 1,075).
Step 2: PPS sampling (10). Hai Chau District has 13 wards, in which each ward had a different total number of married women who had given birth from 4 weeks to 6 months prior to the study. The number of wards selected was 10 (*N* = 10). Sample distance was calculated as *k* = *m*/*N* (1,075/10), so *k* = 108. Random numbers were selected *R* from 1 to *k* (any number); *R* = 30. Clusters to be sampled were selected using cumulative frequencies. Based on the cumulative frequencies, clusters consisting of *R* + *ik* (*i* from 0 to *N* − 1) were sampled. Specifically, they were wards of Thuan Phuoc, Thanh Binh, Thach Thang, Hai Chau 1, Hai Chau 2, Binh Hien, Hoa Thuan Dong, Hoa Thuan Tay, Hoa Cuong Nam, and Hoa Cuong Bac.
  The sample size of each ward was: 600/*t* multiplied by the total number women eligible to participate in that ward (in which *t* is the total number of married women who had given birth from 4 weeks to 6 months in the 10 wards, selected by PP S sampling).
Step 3:Sampling frames were created based on the list of women eligible to participate in each selected ward, SRS was applied to select the number of women in each ward (10).

**Figure 1:**
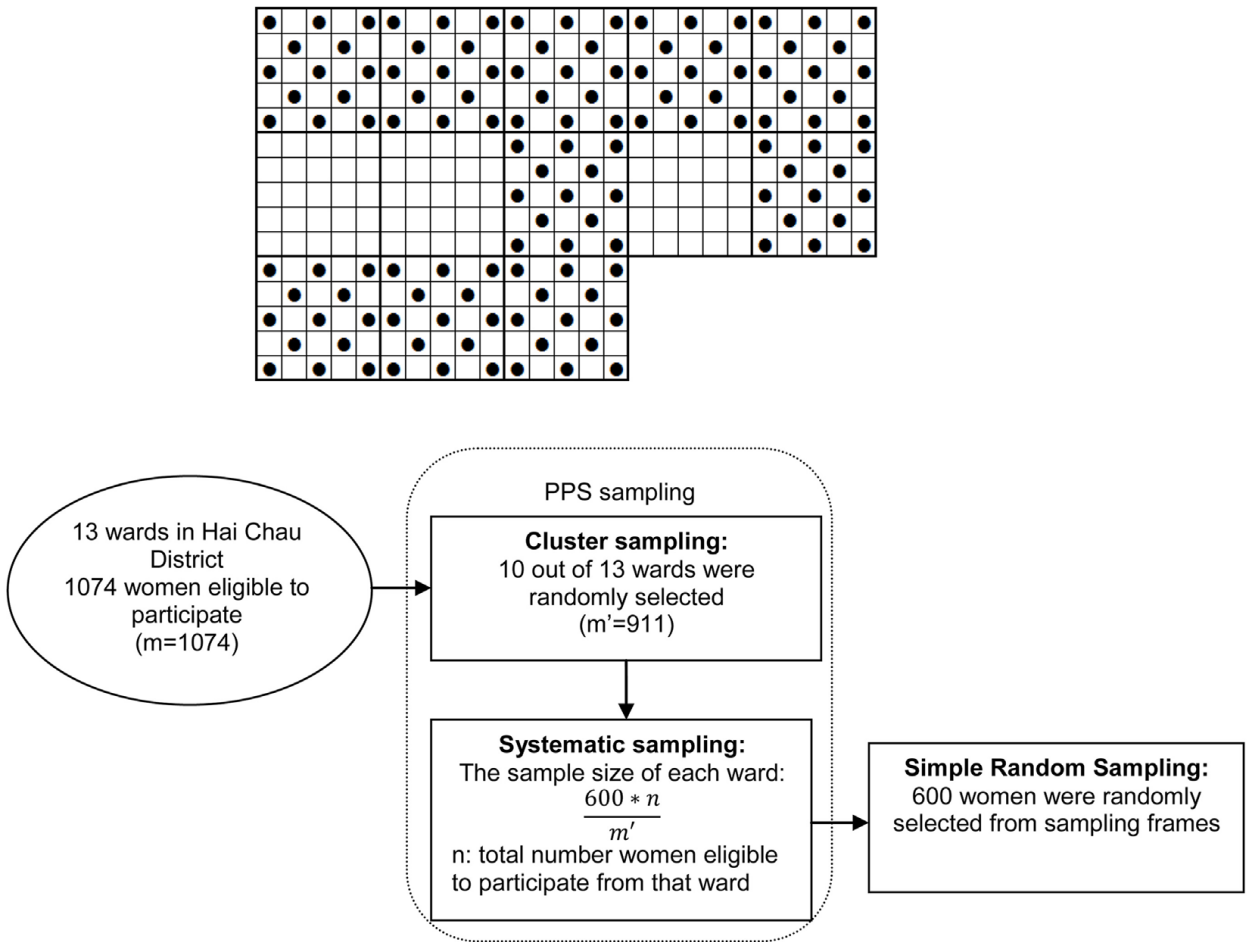
**Two-stage sample (cluster then systematic) in the study**.

The sampling procedure was summarized in Figure 1 as follows.

### Methods of Data Collection

A questionnaire was used to directly interview women from the 10 selected wards of Hai Chau District from July to December 2013. The questionnaire included questions about the respondents’ demographics as follows:

– Age, employment, working conditions, education, and whether their family experienced financial difficulties (defined as earning <VND 9,600,000/person/year);
– Family living arrangements (mother’s economic dependence on husband, living with parents, living with in-laws or renting);
– Expectations regarding the pregnancy; expectation of the gender of the infant; the woman’s relationship with her husband (that the woman could rely on her husband for help, whether she could discuss all problems with her husband, if she felt frightened of her husband in the past year and if she had been beaten by her husband in the past year);
– Exercise after birth; infant illness; and feeling anxiety about matters other than the birth.

The questionnaire was developed by the research team and was validated by a pilot survey. PPD screening was conducted using the Edinburgh Postpartum Depression Scale (EPDS) translated into Vietnamese. The EPDS has been widely used to identify symptoms of PPD and has been verified with a sensitivity of 88% and specificity of 92.5% (11). Most of the studies about PPD conducted in Vietnam apply the EPDS (4). The EPDS questionnaire has 10 items. The mother chooses one of four possible answers to describe what they experienced in the previous week. Each answer is converted to a score on a scale of 0–3. The total possible score is 30. A cutoff point of 12/13 was used to define the presence of PPD symptoms (without PPD symptoms: EPDS ≤12 points/with PPD symptoms: EPDS ≥13) (11).

### Method of Data Analysis

Data were analyzed using the software of SPSS 16.0 Version for Windows. Chi-square tests and multivariate logistic regression using a backward conditional method were undertaken. All variables associated with increased risk of PPD in unadjusted tests were used.

### Resultant Participant Recruitment

Three principles of research ethics were observed throughout this study: respect for privacy, fairness, and benefit to the participants. Before the interview, research purposes were clearly explained. It was also explained that participation was voluntary. The participants were assured that they could stop at any point, if they so desired, and reassured that all responses would be kept confidential.

## Results

### Demographic Characteristics

The mean age of respondents was 28.76 (±4.99), of whom the youngest was 17 and the oldest was 44. The 20–34 age group made up the highest proportion of respondents (84.8%). In terms of employment, the largest single category was public officials, accounting for 36.7%, while the smallest category was manual workers, which accounted for 13.8% of respondents. Overall, 75% of the mothers reported having stable jobs. The research sample consisted of women of all educational levels. Mothers who had less than a secondary level of education accounted for 17.3% of the respondents, including those who had not attended school and those who attended primary or secondary school only. Those with tertiary education and above made up the highest proportion of respondents, at 82.7%, of which 32.7% were graduates or postgraduates.

The proportion of women with financial difficulties within the family was 13.8%. The percentage of women who were economically dependent on their husband or family was 36.5%. Most women lived together with their husband (98.2%), among which the majority lived with their own parents or parents in law (58.7%).

Women who had undertaken a mental health examination during their previous pregnancies or postnatal period had a higher rate of PPD than women who had not had a mental health examination, but this difference was not statistically significant (*p* > 0.05).

### The Prevalence of PPD Symptoms Based on EPDS in Hai Chau District, Danang

The prevalence of PPD symptoms based on an EPDS score ≥13 was 19.3% (95% CI 16.16–22.50) (*n* = 116). Among the 116 women with PPD symptoms, 37.9% reported having suicidal thoughts in the previous 7 days (95% CI: 28.96–46.89).

Table 1 includes all sociodemographic and personal variables of respondents.

**Table 1:** **Demographic characteristics of respondents stratified with postpartum depression status**.

Factors	EPDS score ≥13		EPDS score ≤12		Total
	*n*	%	*n*	%	
**Age groups**
<20	2	16.7	10	83.3	12
20–34	98	19.3	411	80.7	509
≥35	16	20.3	63	79.7	79
Age (mean, SD)	28.67 ± 4.98		28.77 ± 4.99		28.76 ± 4.99
**Employment**
Public officials	31	14.1	189	85.9	220
Manual workers	13	15.7	70	84.3	83
Business	19	18.6	83	81.4	102
Others	53	27.2	142	72.8	195
**The highest attained education**
Junior high school or lower	27	26.0	77	74.0	104
Senior high school	58	19.3	242	80.7	300
Graduates or postgraduates	31	15.8	165	84.2	196
**Marital status**
Living with husband	110	18.7	479	81.3	589
Widowed/separated	6	54.5	5	45.5	11
**Financial difficulties**
Yes	23	27.7	60	72.3	83
No	93	18.0	424	82.0	517
**History of mental health examination in previous pregnancies**
Yes	2	33.3	4	66.7	6
No	114	19.2	480	80.8	594
**Total**	116	19.3	484	80.7	600

### Factors Associated with Postpartum Depressive Symptoms in Hai Chau District, Danang

Table 2 showed the distribution of factors that were associated with PPD symptoms among the respondents.

**Table 2:** **Factors associated with postpartum depressive symptoms (*N* = 600)**.

Factors	EPDS score ≥13		EPDS score ≤12		OR	95% CI
	*n*	%	*n*	%		
**Working conditions**						
Stable	76	16.9	374	83.1	1.79	1.16–2.77
Unstable	40	26.7	110	73.3		
**Women economically dependent on their husbands**						
Yes	53	24.2	166	75.8	0.62	0.41–0.94
No	63	16.5	318	83.5		
**Current place of residence**						
Private house	27	13.8	168	86.2	1.75	1.10–2.80
Parents’ house/rented house	**89**	22.0	**316**	78.0		
**Pregnancy was unexpected**						
Yes	89	17.8	411	82.2	1.71	1.04–2.81
No	27	27.0	73	73.0		
**Baby’s gender was as expected**						
Yes	86	17.5	406	82.5	1.82	1.12–2.94
No	30	27.8	78	72.2		
**Anxious about other matters in addition to giving birth**						
Yes	68	34.2	131	65.8	0.26	0.17–0.40
No	48	12.0	353	88.0		
**Women can rely on their husbands for help**						
Yes	85	16.0	446	84.0	4.28	2.52–7.26
No	31	44.9	38	55.1		
**Women can share all problems with their husbands**						
Yes	82	15.7	440	84.3	4.15	2.50–6.88
No	34	43.6	44	56.4		
**Women felt scared of their husbands during the last 12 months**						
Yes	27	32.1	57	67.9	2.27	1.36–3.79
No	89	17.2	427	82.8		
**Women were beaten by their husbands in the last 12 months**						
Yes	14	38.9	22	61.1	0.35	0.17–0.70
No	102	18.1	462	81.9		
**Women did exercise after giving birth**						
Yes	3	5.0	57	95.0	0.20	0.06–0.65
No	113	20.9	427	79.1		
**Infant illness during the postnatal period**						
Yes	56	35.2	103	64.8	0.29	0.19–0.44
No	60	13.6	381	86.4		
	116		484			

Women’s factors associated with PPD: women’s working conditions, their economic dependence on husbands, current place of residence, pregnancy expectation, expected baby gender, their anxiety about other matters in addition to giving birth, reply on their husbands for help, sharing all problems with their husbands, feeling scared of their husbands during 12 months, violence against women by husbands, exercise after giving birth, and infant illness in postnatal period (*p* < 0.05).

Table 3 continued to show results of multivariate logistic regression analysis to examine factors, after confounding variables were adjusted, associated with PPD based on EPDS.

**Table 3:** **Results of multivariate logistic regression analysis to examine factors associated with postpartum depression based on EPDS**.

Independent variable	Adjusted OR* (95% CI)	*p*
**Women can rely on their husband for help**		
Yes	1	
No	2.26 (1.36–4.81)	0.004
**Women can share all problems with their husbands**		
Yes	1	
No	2.45 (1.35–4.45)	0.003
**Anxious about other matters in addition to giving birth**		
Yes	1	
No	0.33 (0.21–0.52)	<0.001
**Doing exercise after giving birth**		
Yes	1	
No	4.86 (1.44–16.39)	0.011
**Infant illness in postnatal period**		
Yes	1	
No	0.29 (0.18–0.46)	<0.001

*All variables associated with increased risk of PPD in unadjusted tests were analyzed. The multivariate logistic regression model retained five variables in the final step. Adjusted for women can rely on their husband for help, women can share all problems with their husbands, anxious about other matters in addition to giving birth, doing exercise after giving birth, and their infant illness in postnatal period were factors in statistically significant relationship with PPD (*p* < 0.05).

## Discussion

### Prevalence of Postpartum Depressive Symptoms

The prevalence of PPD symptoms within the research population was 19.3%. Other researchers who also used the EPDS and a cutoff point of 13, and conducted their studies on women in various periods within the first year after giving birth, have found differing rates of prevalence. For example, Aydin et al. conducted a study in a Turkish community of women after giving birth from 1 to 12 months and found the PPD prevalence to be 40.1% (4), while research by Pocan et al. (12), also in Turkey, found that the prevalence of PPD in women after giving birth from 4 to 6 weeks was 28.9% (12). In 2010–2011, Murray et al. studied mothers who had given birth from 4 weeks to 6 months in Hue, Vietnam, and found a prevalence of 18.1% (8).

The prevalence found in the study reported here was similar to that found in Murray et al.’s study, and also the findings of a large systematic review of common perinatal mental disorders in low-and-middle-income countries where context-specific as well as gender-sensitive factors considerably contributed to PPD (3). However, it was lower than that found by Fisher et al.’s studies, conducted with mothers after giving birth from 4 to 6 weeks in Ho Chi Minh City in 2004 and in the northern area of Vietnam in 2010, which were 33 and 29.9%, respectively (7, 13). The differences within PPD prevalence may be attributed to many factors. PPD is an illness associated with economic, cultural, and social issues; thus the studies conducted in places with various cultural and social backgrounds will have different rates of PPD. The findings could be influenced by the research design, and the period during which the research was conducted within the first year after giving birth. Moreover, the EPDS as a tool reports prevalence at a point in time rather than incidence of ongoing depression.

### Factors Associated with Postpartum Depressive Symptoms

Our findings indicated that the number of mothers with unstable jobs with symptoms of PPD was higher than that of those who had stable jobs (26.7 vs 16.9%). In addition, the women who were financially dependent on their husbands or families had a higher rate of PPD (24.2%) than those who were financially independent (16.5%). Vietnam has strong policies promoting gender equality, which means both men and women have opportunities for studying and working. However, if a mother did not have stable employment outside of the home, or unemployed or freelances, she could not make financial contributions to the family and thus becomes economically dependent on her husband. Child rearing is a major task, and mothers must spend a number of months caring for their newborn child. Not having independent earnings or paid maternity leave can therefore increase the financial burden on the family. The results of the study here were similar to the study by Trinh (14) and Fisher et al. (7).

Regarding family living arrangements, mothers who lived in rented houses or who were living with their parents had higher rates of symptoms of PPD (22.0%) than those living in their own houses (13.8%). The study by Ekuklu et al. also concluded that living in a rented house was a factor related to PPD [c.f. (4)]. The rapid urbanization of Danang city in recent years has included the building of many industrial parks and has attracted a large number of laborers from rural areas and other provinces. This mass immigration to Danang for living and working has increased the demand for housing and health services among the local people. As a result, mothers living in rented houses will be subjected to the financial pressures of paying rent and/or be concerned that the house lacks basic amenities, hygiene, and security. These factors may affect the family’s living standards, which may partially explain this association with increased PPD symptoms in mothers who rent.

The results also found that mothers who had an unexpected pregnancy were more likely to have symptoms of PPD than that of those who had an expected pregnancy (27.0% compared with 17.8%). This result was consistent with the findings of Klainin and Arthur (4), which reported that unexpected pregnancy was meaningfully related to PPD in 22 countries from Asia (4). With an unexpected pregnancy, the mother may not have time, support, or resources to prepare spiritually, physically, and economically to have a new baby.

Expectations of infant gender were also associated with symptoms of PPD. In all, 27.8% of the mothers with PPD symptoms did not expect the gender of their baby (primarily, those who hoped for baby boy but gave birth to baby girl). The prevalence of PPD symptoms in these mothers was higher than those with an infant whose gender was expected (17.5%). These results were similar to the study by Klainin and Arthur (4), which synthesized seven studies in Asia and recognized the connection between unexpected gender and PPD (4). In Vietnam, the preference for sons over daughters originates from Confucianism and patriarchal societal norms, where property and family names were inherited through males (8). Similarly, a study by Thanh et al. (15) revealed that 6.2% of women were forced to divorce by their husband’s family because they were unable to deliver a baby boy (15). Murray et al. (8) found that while having an infant that was not the gender that was expected affected maternal wellbeing, it was not significantly associated with postpartum depressive symptoms. The inconsistent findings about this association from a number of studies indicate that more research into the effect of son preference and family planning policies on perinatal mental health is needed in Vietnam.

Our results indicate that women who were anxious about matters other than giving birth had a higher rate of PPD (34.2%) than those without anxiety (12.0%). Siu et al. (16) claimed that stresses in life were meaningfully connected to PPD (RR = 2.56; 95% CI: 1.84–3.57; *p* < 0.001) (16).

Regarding women’s relationships with their husbands, the analysis showed that women who did not get support from their husband had a higher rate of PPD (44.9%) than those who had support from their husband (16.0%). As shown in the multivariate logistic regression analysis in Table 3, women who were not helped by their husband were twice as likely to have symptoms of PPD compared to those who relied on their husband (OR = 2.26; 95% CI: 1.36–4.81). This result was similar to those found in studies by Fisher et al. (7, 13). After giving birth, a woman needs a lot support from her husband and family. Not having time to share their problems with their husband was a factor related to PPD (OR = 2.45; 95% CI: 1.35–4.45). This finding was consistent with other studies by Trinh (14) who reported that pregnant women who can confide in their husbands will have a reduced risk of being affected by PPD (14).

Thirty-two percent of women who felt afraid of their husband in the first year post-childbirth had symptoms of PPD, which was associated with higher EPDS scores. In addition, there was a difference in the prevalence PPD symptoms among women who were hit by their husbands (38.9%) compared to those who did not experience being hit (18.1%). This result was similar to that of the study by Murray et al. (8) and reveals the close connection between women who were afraid of their husband in the previous year and the average EPDS (*p* < 0.001) (8). Similarly, the study by Fisher et al. (3) concludes that domestic violence was a social factor that was strongly associated with PPD in low-and-middle-income countries, including Vietnam (3). In Danang, according to the statistics of the Department of Culture, Sports and Tourism in 2014, the victims of domestic violence were mostly women, with the main causes being attitudes related to feudal ideology, family’s financial problems, alcohol abuse, controlling behavior, and a lack of legal knowledge (17). The results of our study were also consistent with those found by Woolhouse et al. (18) and Pocan et al. (12): there was a strong relation between domestic violence and PPD (12, 18).

Physical health during the postpartum period was also relevant to the findings. We found that women who exercised were less likely to have PPD symptoms than those who did not (20.9 vs 5.0%, respectively). Our study concluded that not exercising after giving birth was a factor related to PPD symptoms (OR = 4.86; 95% CI: 1.44–16.39). There was evidence that postnatal exercise can help women recover postpartum, regain strength, and reduce stress. Diem and Nguyen (19) suggest that exercise helps to produce and increase serotonin in the brain, which regulates mood, and will make one feel more relaxed and comfortable (19).

Another important association with symptoms of PPD was having an infant who was sick in the period between giving birth and the time of the interview. Mothers with sick infants were more likely to have PPD symptoms than mothers whose infants were healthy (35.2 vs 13.6%). The multivariate regression analysis also indicates that having a sick newborn was a factor related to PPD (OR = 0.29; 95% CI: 0.18–0.46). Clearly, the health of the infant can have a considerable effect on their mother’s mood. With respect to this factor, the findings were similar to studies by Xuan Đen (Ho Chi Minh City), Glasser (Israel), Manl, KD (USA) (4, 15, 20).

We finally only five factors were significantly associated with PPD symptoms after multivariate logistic regression analysis (*p* < 0.05). These five factors included: women can rely on their husband for help, women who can share all problems with their husbands, anxious about other matters in addition to giving birth, doing exercise after giving birth and having a sick infant in the postnatal period. In Vietnam, especially in coastal provinces in the central region as Danang city, unequal treatment of individuals based on their gender is a deeply rooted problem. Such attitudes, together with the impacts of modern lifestyles may influence the prevalence of PPD.

## Limitations

This study only used the EPDS to assess postpartum depressive symptoms, thus it was quantitative research providing an estimate of symptoms rather than clinical diagnoses of depression. The social and cultural variables included were based on existing literature. However, a qualitative approach may have revealed more about the subjective factors associated with postpartum depressive symptoms, such as opportunities for social support gained through connection with societies like the women’s union or association of other new mothers. Only married women were included in the sample. Although the number of unmarried mothers in Vietnam is very small, these women might indeed be at high risk for PPD. The study population was located near Danang, which was one of the fourth largest cities in Vietnam. Factors associated with PPD in Danang might be different for women in smaller towns or rural areas. Finally, as the study was cross-sectional; the duration of PPD symptoms, and the relationship between PPD and antenatal depression, was not studied.

## Conclusion

The prevalence of postpartum depressive symptoms was 19.3%, which was comparable with other prevalence estimated from Vietnam. After adjusting for other variables in a multivariate logistic regression analysis, the variables associated with postpartum depressive symptoms included reliance on the husband; not being able to discuss problems with their husband; being anxious about matters in addition to giving birth; not exercising after giving birth, and having an ill baby in the postnatal period. The prevalence of PPD symptoms and the factors related to PPD (relationships with husbands and family, exercise, and infant health) should be considered when developing preventive, diagnostic, and therapeutic programs that will promote the long-term health of mothers and their newborns.

In addition, it is important to conduct further research to investigate programs aimed at women at higher risk of perinatal mental illness. Early screening procedures in primary care services may be useful for identifying families in need of more specialized mental health care among pregnant women.

## Ethics Statement

This study was approved by the ethics review panel (Institutional Review Board of Hue University of Medicine and Pharmacy). Respondents were informed about the study, invited to participate, and asked to sign an informed consent form. Of the 600 identified for the sample of the study, 600 agreed to participate, for a response rate of 100%.

## Author Contributions

TV was the person who was responsible to supervise HD doing this research as her Master of public health thesis and writing this manuscript. HD was MPH student who was responsible developing her research proposal and finishing her thesis. She also wrote this manuscript under TV’s supervision. TH contributed to this manuscript as supporting data analysis at high level using statistical software of SPSS.

## Conflict of Interest Statement

The authors declare that the research was conducted in the absence of any commercial or financial relationships that could be construed as a potential conflict of interest.
